# Association between night shift work and risk of osteoporosis and osteoporosis-related pathological fracture

**DOI:** 10.3389/fpubh.2025.1719807

**Published:** 2026-01-13

**Authors:** Daishui Yang, Cheng Xiang, Tianyu Wang, Xiaoning Guo, Zhengxiao Ouyang, Lu Wang

**Affiliations:** Department of Orthopedics, The Second Xiangya Hospital, Central South University, Changsha, Hunan, China

**Keywords:** osteoporosis, fracture, night shift work, genetic risk, UK biobank

## Abstract

**Background:**

Night shift work may increase the risk of various diseases, this study aims to explore the associations of both past and current night shift rotations with osteoporosis and its related fracture risks, and to evaluate the role of potential mediators further in the UK biobank.

**Methods:**

We investigated 276,774 UK Biobank participants with current paid employment or self-employed work, alongside 75,120 individuals with lifetime occupational history information. The multivariable-adjusted logistic regression and Cox proportional hazard models were utilized to analyze the potential links between night shift work and the incidence of OP. Moreover, stratified analyses and interaction tests were applied to explore potential modifications.

**Results:**

A total of 276,774 participants aged 40 to 69 years (mean ± SD: 52.7 ± 7.1); among them, 143,081 women (51.7%); 260,994 White participants (94.3%) were included, and a total of 5,906 OP events were documented. Relative to “day workers,” all subgroups engaged in night shifts displayed a notable increase in OP prevalence (*P* for trend < 0.001). The usual night shifts with the highest risk [hazard ratio (HR) 1.29, 95% confidence interval (CI) 1.12–1.50]. Besides, the results of lifetime night shift schedules showed the highest OP risk participants with duration >10 years [OR 1.21, 95% CI (0.92–1.58)] and 3–8 shifts/month [OR 1.38, 95% CI (1.11–1.72)] exposure. In addition, these positive associations remained unaltered by sensitivity and stratified analyses with various confounders and genetic vulnerability (all *P* for interaction > 0.05). Furthermore, we observed a positive association between night shifts and OP-related pathological fractures [HR 1.88, 95% CI (1.20–3.94)].

**Conclusion:**

This study demonstrated that both current and lifetime night shifts was significant associated with the greater risk of OP and its related pathological fracture. Additionally, these associations were not modified by genetic susceptibility. The potential of reducing night shift work as a strategy for preventing osteoporosis requires further investigation.

## Introduction

Osteoporosis (OP) stands as a pervasive systemic skeletal disorder, commanding global attention as a significant public health issue (1). The National Center for Health Statistics (NCHS) has reported that over half of adults aged 50 and above in the United States either grapple with OP or confront an elevated vulnerability to its onset due to progressive bone mass reduction (2). The American Academy of Orthopedic Surgeons (AAOS) underscores the potential of robust primary prevention in curtailing OP incidence, encompassing interventions like physical activity, lifestyle adjustments, and, in certain cases, orthopedic medications to alleviate the decline in bone mineral density (3). Therefore, new common risk factors for osteoporosis are crucial to be actively investigated.

Human physiological functions are primarily orchestrated by an inherent circadian rhythm, orchestrating the optimization of biological processes in sync with daily environmental and behavioral patterns (4). Shift work, characterized by employment outside conventional daytime hours, stands as a significant disruptor of circadian rhythms and sleep cycles (5). Prolonged and frequent engagement in night shift work further holds the potential to perturb metabolic functions and disrupt hormonal secretion, thereby elevating susceptibility to chronic ailments (6). This shift work trend has gained traction within industrialized nations. A survey on working conditions across the Western hemisphere indicates that approximately 21% of European Union employees and 29% in the United States are immersed in shift work arrangements (7). In recent years, numerous researches indicated that night shifts could increase the risk of disease incidence and health damage, such as obesity (8, 9), healthy aging (10) and coronary heart disease (11, 12), only several small-scale investigations evidence involved OP, and the correlation between night shift work and OP has remained inconclusive (13–17).

In this study, a comprehensive cohort of approximately 280,000 individuals was involved. Our primary research objective is to analyze the potential linkage between current night shift work and an elevated risk of developing OP. Leveraging data encompassing lifetime employment details, we delved into whether the duration and frequency of night shift work exhibit a correlation with a higher risk of OP. Furthermore, an exploration into the interplay between night shift work and genetic predisposition to OP was undertaken to ascertain its impact on disease susceptibility. As a secondary investigative aim, we also evaluated the association between night shift work and the risk of OP-related pathological fractures.

## Methods

### Study design and participants

During the period of 2006 to 2010, a prospective cohort study in the United Kingdom (UK), UK biobank, enlisted over half a million individuals. The participants, spanning ages 37 to 73, data compilation encompassing a spectrum of facets, spanning lifestyle, mental well-being, physical metrics, biological evaluations, and genomic information (18, 19). Ethical approval for the study was granted by the North West Multicenter Research Ethics Committee, and all participants rendered written informed consent. For access to the original data, please refer to the UK Biobank website^1^.

For the scope of this study, our focus was directed toward participants who were engaged in paid employment or were self-employed at the time of recruitment and with complete data of night shift exposure and important covariates. Accordingly, a total of 276,774 participants were included in the main analysis. In the prospective study within the current night shift cohorts, individuals with a pre-existing diagnosis of osteoporosis were excluded from consideration (*N* = 3,052). In addition, the UK Biobank program conducted an online lifetime employment questionnaire follow-up with approximately 330,000 participants who had provided contactable email addresses, regardless of their work status at the baseline assessment (11). Among them, a subset was conformed to our inclusion criteria (*N* = 75,120). The additional questionnaire provided detailed information on the cumulative duration and average frequency of night shift work before the baseline. The visual representation of our research protocol is presented in Supplementary Figure 1.

### Shift work assessment

During the baseline assessment, all participants engaged in paid or self-employed positions were queried about their prevailing work schedules. This led to the classification of participants into two categories: day workers, adhering to the standard 9 a.m. to 5 p.m. timetable, and shift workers, who had work schedules outside of this timeframe, including afternoon, evening, night, or mixed rotation shifts. The primary focus of this study was directed toward individuals actively involved in night shift work, which was defined as employment during the conventional sleep hours, specifically spanning at least 3 h between 12 a.m. and 6 a.m. Response options included ‘never/rarely’, ‘sometimes’, ‘usually’, and ‘always’. Aligning with previous research for consistency, participants were categorized into four distinct categories: ‘day workers’, ‘shift but never/rarely night shifts’, ‘some night shifts’, and ‘usual/permanent night shifts’ (20).

The in-depth collection of all paid jobs was systematically done through the web-based questionnaire. Except for each job ever worked, participants’ exposure to night shifts was well recorded. This includes instances with no night shifts, a mixture of day and night shifts, and instances with only night shifts. Furthermore, the start and end dates of each job year were recorded in an editable ‘career timeline.’ The information regarding night shift exposure prior to the baseline was extracted for each participant. Utilizing the aforementioned data, the cumulative duration and average frequency of night shifts worked in the past were calculated.

### Assessment of outcomes

The primary outcome measure undertaken in this study was OP, further categorized into three distinct subtypes as per the 10th Revision of the International Classification of Diseases (ICD-10). These subtypes encompassed OP with pathological fracture (field 131,963, ICD code M80), OP without pathological fracture (field 131,965, ICD code M81), and OP in diseases classified elsewhere (field 131,967, ICD code M82) (21, 22). The requisite information pertaining to these classifications was drawn from national registers inclusive of the death register, primary care records, and hospital inpatient records. Additional comprehensive insights regarding this classification process can be accessed online through the following link^2^. Moreover, another pivotal outcome encompassed the OP with pathological fracture only.

### Genetic risk score

The genotyping data utilized within this study were derived from the Genomics PLC of UKB-PRET^3^. Detailed methodologies pertaining to the genotyping procedures have been documented in prior records (23). Briefly, the polygenic risk scores (PRS) in the UK Biobank were constructed using a Bayesian method applied to meta-analyzed GWAS summary statistics. The PRS were generated either entirely from external GWAS datasets (Standard PRS) or from a combination of external and UK Biobank internal data (Enhanced PRS). To reduce ancestry-related distributional biases, scores underwent principal component–based ancestry centering and were subsequently standardized to have approximately unit variance within ancestry groups. In our study, the Standard PRS for osteoporosis (OP) was calculated for 486,128 participants and used for analysis. This PRS was derived from a set of independent single nucleotide polymorphisms (SNPs), gleaned from the GWAS training data, and was computed through a weighted approach (24). The calculation of PRS involved summing the products of the SNP values (SNP1, SNP2, …, SNPn) and their corresponding beta coefficients (β1, β2, …, βn), and dividing the sum by the total number of SNPs. The beta coefficients represent the association sizes (25). In line with previous studies, participants in our study were categorized into three levels of genetic risk for OP: low, intermediate, and high (26).

### Covariates

The covariates encompassed within this study were organized into three groups. Firstly, demographic variables were considered, including age (at baseline), sex (male/female), ethnicity (white/others), educational attainment (university or college degree/others), body mass index (BMI), and the Townsend deprivation index, serving as a proxy for socioeconomic status.

The second group included four main parameters germane to OP, as defined by the AAOS: physical activity, smoking status, alcohol status, and healthy diet. To be specific, data pertaining to physical activity were sourced from the International Physical Activity Questionnaire short form, accounting for moderate activities ≥150 min/week and/or vigorous activity ≥75 min/week, or equivalent combination aerobic according to the activity guideline of the UK (27), quantified in metabolic equivalent task (MET-hours/week). Smoking and alcohol status details were derived from self-administered touchscreen questionnaires, categorizing smoking status as ‘never,’ ‘previous,’ and ‘current’. Information on a healthy diet, encompassing vegetable intake (both cooked and raw), fruit consumption (both fresh and dried), fish consumption (both oily and non-oily), processed meat intake, and unprocessed meat intake (including poultry, beef, lamb, and pork), was obtained through self-report questionnaires (28).

Thirdly, an evaluation of sleep chronotype and duration was undertaken through self-reported sleep questionnaires. Sleep chronotype was stratified into three categories: ‘morning type’ (a ‘morning’ person), ‘intermediate type’ (more of a ‘morning’ than ‘evening’ person, or more of an ‘evening’ than ‘morning’ person), and ‘evening type’ (an ‘evening’ person), aligning with previous research (29). Furthermore, our study also incorporated information on chronic diseases (diabetes) and cancer, as well as the use of vitamins (specifically vitamin D) and mineral and other dietary supplements (including fish oil, glucosamine, and calcium). Additionally, the duration of outdoor activities during both summer and winter was considered.

### Statistical analyses

All statistical analyses were carried out using the IBM SPSS Statistics, version 27 (IBM Corporation, Armonk, NY, United States) with the built-in R package (30). Continuous variables were represented as mean ± standard deviation, while categorical variables were expressed as percentages. A significance level of *p* < 0.05 was deemed indicative of statistical significance.

We examined the associations of current night shift status (‘day workers’, ‘rarely night shift’, ‘some night shift’ and ‘usual night shift’) with the risk of osteoporosis incident. The calculation of follow-up time for each participant commenced from the assessment date (date of attending the assessment center) and culminated at the cessation date (marked by an OP diagnosis, mortality, or the expiration date), or October 31, 2022, whichever came first. The Cox proportional hazard models were used to calculate the hazard ratios (HR) and 95% confidence intervals (CI) in the association between current night shift and incident osteoporosis. For lifetime night shift exposure, we explored the association of cumulative duration of night shifts (‘none,’ ‘<5 years,’ ‘5–10 years,’ and ‘>10 years’) and average monthly frequency of night shifts (‘none,’ ‘<3/months’, ‘3–8/months’ and ‘>8/months’) with osteoporosis odds. The multivariable-adjusted logistic regression models were utilized to examine the odds ratio (OR) and 95% CI of night shift with osteoporosis at the baseline.

For the regression models, Model 1 entailed adjustment for age, sex, and BMI. Model 2 extended the adjustments to encompass ethnicity, education, and the Townsend index. Model 3 encompassed all variables adjusted in Model 2, incorporating AAOS OP-relevant variables. The reference groups for current and lifetime work status were defined as ‘Day workers’ and ‘no night shift worked’, respectively. Trend tests (*P* for trend) were executed with continuous values, extracted from categorical variables including current work status, lifetime duration and frequency.

Several sensitive analyses were conducted in our study: (1) excluded participants who occurred osteoporosis within the first 2 years after the recruitment; (2) the multiple imputation method was used to deal with the missing value; (3) adjust for other chronic disease (diabetes) and cancer; (4) further adjust for sleep duration and sleep chronotype; (5) further adjust for spend time outdoor in summer and winter; (6) further adjust for vitamin and mineral supplements; (7) In female-specific group, additional adjust for menopause and hormones use. Furthermore, we performed stratified analyses to assess the consistent of the relationship between night shift work and OP incident risk, with variables such as sex, sleep duration, chronotype, and AAOS OP-related confounders, based on a log-likelihood ratio test comparing models with and without cross-product interaction terms. Finally, we aimed to ascertain whether genetic predisposition to OP could potentially modulate the interaction between night shift work and the risk of OP incidence, spanning both current and lifetime work statuses. The exploration of the interplay between the continuous PRS and tertile-categorized PRS with OP involved the use of a multivariable-adjusted regressive model. Stratified analysis was then applied using PRS categories and the categories of current employment and lifetime employed night shift work (4). Additionally, we also investigated the association between current night shifts and OP-related pathological fractures as a secondary outcome.

## Results

Table 1 presents the overview of baseline characteristics observed in this study. Post the application of screening criteria, the final 276,774 participants within 5,906 occurrences of OP events were documented over the follow-up period. The work schedule distribution in the four groups (‘day workers,’ ‘shift but never/rarely night shifts,’ ‘some night shifts,’ and ‘usual/permanent night shifts’) was as follows: 82.7, 8.5, 4.9, and 3.8%, respectively. In comparison to the ‘day workers’ group, the night shift workers displayed a propensity toward being male, relatively younger, with lower educational qualifications, longer work durations, and a higher incidence of socio-economic disadvantage. This cohort was also characterized by a higher proportion of non-European ethnicities, higher BMI and elevated diabetes prevalence, as well as shorter sleep durations and a proclivity for later chronotypes. Moreover, a comprehensive analysis was also performed to capture the population characteristics stratified based on lifetime night shift duration and average monthly night shift frequency. These details are further elaborated in the Supplementary Tables S1, S2, S7.

**Table 1:** UK Biobank participants’ characteristics by current work exposure (*n* = 276,774).

Characteristics	Current work schedule			
	Day workers	Rarely night shifts	Some night shifts	Usual night shifts
*N* (%)	228,971 (82.7)	23,502 (8.5)	13,677 (4.9)	10,624 (3.8)
Age (years)	52.9 (7.1)	52.5 (7.0)	51.2 (6.8)	51.3 (6.8)
Sex (male, %)	46.9	47.7	62.3	62.4
BMI (kg/m^2^)	27.1 (4.6)	27.8 (5.0)	28.2 (4.9)	28.4 (4.9)
Ethnicity (white, %)	95.3	91.1	88.3	87.2
Education (university or college, %)	41.3	25.1	24.2	15.6
Townsend deprivation index	−1.5 (2.9)	−0.6 (3.2)	−0.6 (3.3)	−0.4 (3.3)
Sleep duration (%) (h/day)	7.1 (1.0)	7.0 (1.2)	6.9 (1.2)	6.7 (1.4)
Chronotype (evening, %)	8.9	8.8	10.7	17.5
Smoke				
Never	58.3	53.8	52.8	52.7
Previous	32.0	32.4	30.9	30.3
Current	9.7	13.8	16.3	17.0
Alcohol				
Never	3.0	4.7	4.6	6.0
Previous	2.5	3.5	3.3	3.7
Current	94.5	91.8	92.2	90.3
Physical activity at goal				
No	18.1	13.9	12.1	11.6
Yes	66.1	65.0	67.3	64.7
Missing	15.8	21.1	20.5	23.7
Healthy diet				
Vegetable (tablespoons/day)	4.8 (3.2)	4.9 (3.6)	4.9 (3.9)	4.8 (3.9)
Fruit (tablespoons/day)	3.0 (2.4)	2.9 (2.6)	2.9 (2.7)	2.9 (2.9)
Fish (times/week)	2.2 (1.5)	2.2 (1.6)	2.3 (1.8)	2.2 (1.7)
Processed meat (times/week)	1.5 (1.4)	1.6 (1.5)	1.7 (1.5)	1.7 (1.5)
Unprocessed meat (times/week)	4.0 (2.1)	4.1 (2.2)	4.4 (2.3)	4.5 (2.4)

Continuous variables are displayed as means (SD), and categorical variables are presented as percentages.

The association between current work status and OP risk was first examined (Table 2). In the Cox proportional-hazards model 1 adjusted for age, sex and BMI, found a notable trend: as the categories of night shifts increased, so did the association with the risk of OP incident (*P* for trend <0.001). Moreover, the usual night shift workers demonstrated the highest risk [HR 1.29, 95% CI (1.12–1.50)]. Subsequently, multivariable adjusted models of model 2 and model 3 were conducted, substantiated the persistently positive trend (both *P* for trend < 0.001), although the strength of the association was slightly attenuated. When further restricting individuals with osteoporosis incident ≥ 2 years from the recruitment, the above positive association was more notable (Supplementary Table S3). In addition, the exploration of the link between OP-related pathological fracture and night shift work, the results indicated a significant association between “Usual night shift” and an increased risk of fracture incidents, this positive association persisted in the most adjusted model. [HR 1.87, 95% CI (1.20–2.94)] (Supplementary Table S4).

**Table 2:** Association between current shift work and osteoporosis in the UK Biobank (*n* = 276,774).

Variable	Current work schedule				*P* for trend
	Day workers	Rarely night shifts	Some night shifts	Usual night shifts	
Total cases	4,966	526	223	191	
Total sample size	228,971	23,502	13,677	10,624	
Model 1	1.00 [reference]	1.12 (1.02–1.23)	1.19 (1.04–1.36)	1.29 (1.12–1.50)	<0.001
Model 2	1.00 [reference]	1.09 (1.00–1.20)	1.17 (1.02–1.33)	1.26 (1.09–1.46)	<0.001
Model 3	1.00 [reference]	1.09 (0.99–1.20)	1.13 (0.98–1.30)	1.25 (1.07–1.45)	<0.001

Model 1 adjusted for age, sex and BMI.

Model 2 additionally adjusted for ethnicity (white, others), education (university or college, others) and Townsend deprivation index.

Model 3 additionally adjusted for smoke (never, previous, current), alcohol (never, previous, current), physical activity at goal (no, yes, missing), healthy diet (vegetable, fruit, fish, unprocessed meat, processed meat).

Subsequently, the investigation extended to the relationship between work involved rotating night shifts before baseline and OP incident, encompassing a cohort of 75,120 participants and 806 OP cases at baseline (Tables 3, 4). Inclusive analyses of cumulative duration and average frequency of lifetime night shift work revealed positive associations with the OP odds. In the age-, sex-, and BMI-adjusted model, participants who worked schedules involving night shifts for <5 years had a higher likelihood of osteoporosis (OP) than those who never worked night shifts [OR 1.22, 95% CI (0.95–1.57)]. However, multivariable adjustment model strengthened the association between longer night shift exposure and a higher risk of OP incidents (*P* for trend 0.063), in the ‘>10 years’ night shift exposure group have the highest OP risk [OR 1.21, 95% CI (0.92–1.58)]. The average frequency of night shift in the past was associated with OP odds in model 1 (*P* for trend 0.044). After adjustment for other confounders in model 3, this association was still robust (*P* for trend 0.049). In addition, participants who worked an average of 3–8 nights per month, rather than >8 nights per month, had a notable and higher risk of OP than those who never worked night shifts [OR 1.38, 95% CI (1.11–1.72)]. In further sensitivity analysis, imputation of missing values (Supplementary Tables S5, S6), adjustment for chronic diseases (diabetes) and cancer (Supplementary Tables S8, S9, model I), sleep duration, and chronotype (Supplementary Tables S8, S9, model II), spending time outdoors in summer and winter (Supplementary Tables S8, S9, model III), and mineral and vitamin supplements (Supplementary Tables S8, S9, model IV) from the baseline did not substantially modify the existing association. In addition, after adjusting for female-specific variables like menopause and hormone use in the female cohorts, the above association was still notable (Supplementary Tables S10, S11). These findings collectively bolster the evidence supporting the positive connections between current and past night shift work and the risk of OP incident.

**Table 3:** Lifetime night shifts cumulative duration and osteoporosis incidence at the baseline (*n* = 75,120).

Variable	Lifetime duration of night shift work				*P* for trend
	None	<5 years	5–10 years	>10 years	
Total cases	637	68	40	61	
Total sample size	57,200	6,914	3,883	7,123	
Model 1	1.00 [reference]	1.22 (0.95–1.57)	1.18 (0.85–1.64)	1.18 (0.91–1.55)	0.082
Model 2	1.00 [reference]	1.20 (0.93–1.55)	1.18 (0.85–1.63)	1.18 (0.90–1.55)	0.090
Model 3	1.00 [reference]	1.19 (0.92–1.54)	1.21 (0.87–1.67)	1.21 (0.92–1.58)	0.063

Model 1 adjusted for age, sex and BMI.

Model 2 additionally adjusted for ethnicity (white, others), education (university or college, others) and Townsend deprivation index.

Model 3 additionally adjusted for smoke (never, previous, current), alcohol (never, previous, current), physical activity at goal (no, yes, missing), healthy diet (vegetable, fruit, fish, unprocessed meat, processed meat).

**Table 4:** Average frequency of lifetime night shift worked and osteoporosis risk at the baseline (*n* = 75,120).

Variable	Average lifetime night shift frequency				*P* for trend
	None	<3/ months	3–8/months	>8/months	
Total cases	637	17	98	54	
Total sample size	57,200	2,386	8,946	6,588	
Model 1	1.00 [reference]	0.84 (0.52–1.37)	1.37 (1.10–1.70)	1.10 (0.83–1.45)	0.044
Model 2	1.00 [reference]	0.83 (0.51–1.35)	1.37 (1.10–1.70)	1.08 (0.81–1.43)	0.055
Model 3	1.00 [reference]	0.85 (0.52–1.38)	1.38 (1.11–1.72)	1.08 (0.81–1.44)	0.049

Model 1 adjusted for age, sex and BMI.

Model 2 additionally adjusted for ethnicity (white, others), education (university or college, others) and Townsend deprivation index.

Model 3 additionally adjusted for smoke (never, previous, current), alcohol (never, previous, current), physical activity at goal (no, yes, missing), healthy diet (vegetable, fruit, fish, unprocessed meat, processed meat).

Additionally, we performed stratified analyses to assess the consistent of the relationship between night shift work and the occurrence of OP, while considering variables such as sex, sleep duration, chronotype, and AAOS OP-related confounders. The overall findings indicated non-significant interaction effects across the above variables and current night shift status on osteoporosis risk: sex (*P*_interaction_ = 0.674), BMI (*P*_interaction_ = 0.614), sleep chronotype (*P*_interaction_ = 0.156), sleep duration (*P*_interaction_ = 0.350), etc. (Supplementary Table S12). Similarly, there was no notable interaction effect observed between the above variables and lifetime night shift (cumulative duration and average frequency) on OP likelihood (Supplementary Tables S13, S14). In summary, these findings suggests that the associations between both current and lifetime night shift status and the risk of OP remained consistent irrespective of variations in sleep chronotype, sleep duration or other AAOS suggested indexes.

**Figure 1:**
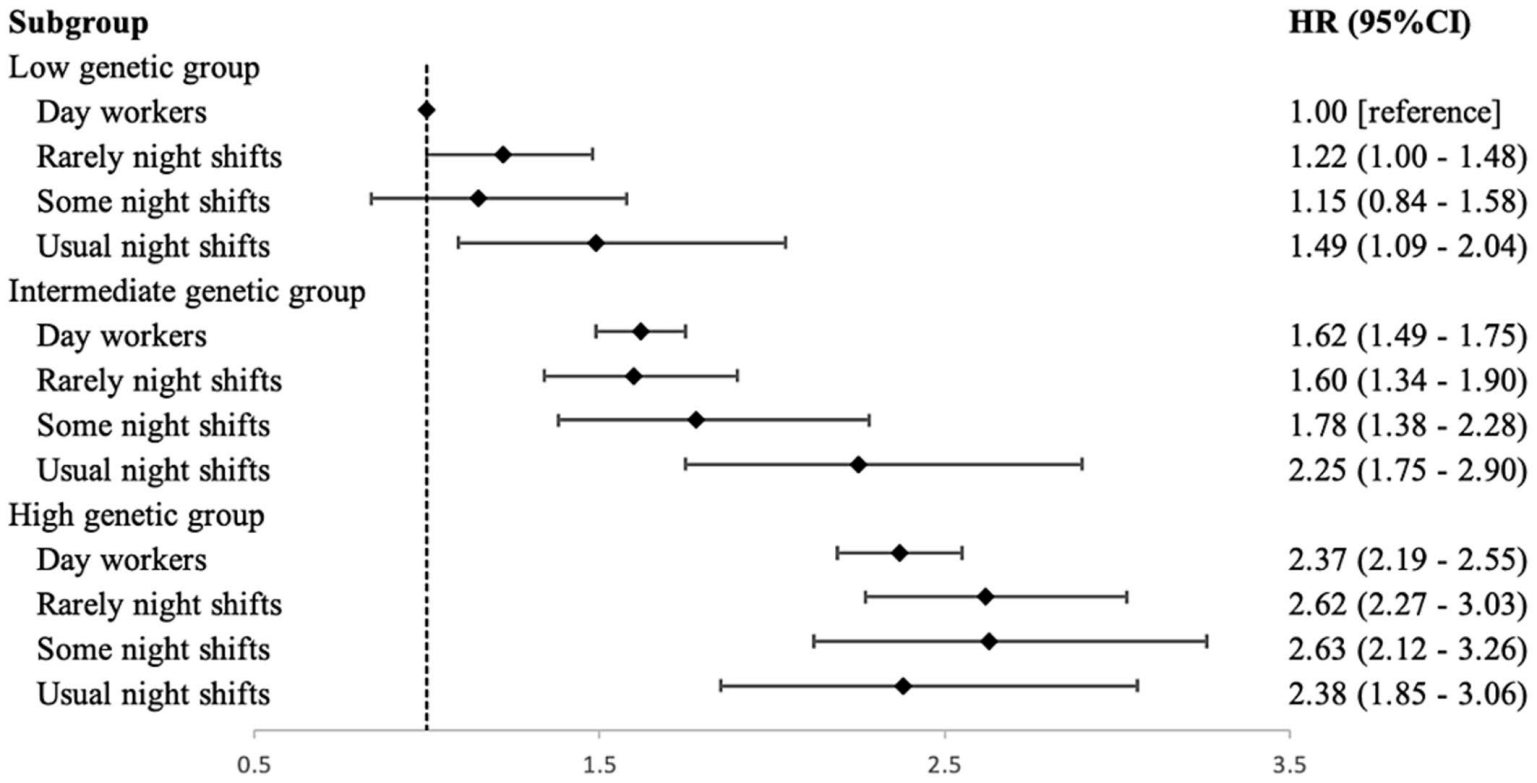
Joint effect of current night shift and PRS on the risk of incident osteoporosis (*n* = 270,483).

Furthermore, we embarked on an exploration into whether the genetic susceptibility of OP could potentially modify the association between current night shift work and the risk of OP. Our findings demonstrated a positive correlation between the Polygenic Risk Score (PRS) and OP incidents. Individuals were categorized into tertiles groups based on their PRS scores, and exhibited an escalating risk of OP occurrence. Specifically, the intermediate [HR 1.58, 95% CI (1.47–1.70)] and high PRS groups [HR 2.31, 95% CI (2.15–2.48)] displayed higher ratios of OP occurrence in comparison to the low PRS group (Supplementary Table S15). When factoring in the current night shift status and tertiles of PRS, we conducted across a total of 12 subgroups. Reference to the group of ‘low genetic risk and day workers’, participants with high genetic risk engaged in ‘Some night shifts’ exhibited the highest risk of OP [HR 2.63, 95% CI (2.12–3.26)] (Figure 1). A similar pattern emerged for subgroups categorized by lifetime cumulative duration (5–10 years), average monthly frequency (3–8/month), indicating the highest OP risk in the high genetic risk groups [OR 3.43, 95% CI (2.16–5.46)], [OR 3.67, 95% CI (2.63–5.12)], respectively. Moreover, our analyses did not reveal any significant interaction effects between genetic susceptibility and the categories of current night shifts (*P* interaction = 0.280), lifetime duration (*P* interaction = 0.601) and monthly frequency (*P* interaction = 0.424), on the risk of OP (Supplementary Tables S16–S18).

## Discussion

In this large prospective study of over 280,000 individuals with about 6,000 OP cases in the UK Biobank, we observed that (1) current night shift workers showed an increased susceptibility to OP compared to those “day worker” after adjusting for other established risk factors. As the categories of night shift work increased (from “rare night shift” to “permanent night shift”), the trend in the incidence of OP became more pronounced; (2) Individuals exposed to night shift work for a lifetime duration exceeding 10 years or 3–8 per month shifts faced higher risk of developing OP; (3) no significant interaction was observed between night shift work in current and lifetime and genetic susceptibility to OP; (4) Besides, within current night shift cohort, the risk of suffering from OP with fractures appeared to be more prevalent in usually night shift worker.

Several studies have explored the effect of exposure to night shift work on OP and bone mineral density. However, these findings are inconsistent. Quevedo and Zuniga (15) were the first to conduct research in this aspect, revealing that long-term night shift work among 70 nurses resulted in an increased risk of OP. Subsequent research by Kim et al. (17) was consistent with this finding, which results indicated that shift of work was linked to higher risk for osteopenia in 3005 Korean residents. Moreover, Feskanich et al. (31) assessed over 30,000 nursing staff and discovered that night shift work significantly increased the risk of fractures in the hip and wrists. In contrast, recent studies conducted by Bukowska-Damska et al. (14) and Santhanam et al. (16), involving a total of over 1,000 participants, demonstrated that night shift work did not link to a significant decrease in bone mineral density. The inconsistent findings may primarily be attributed to factors such as difference in sample sizes and study design, inconsistencies in the collection of demographic information, and variations in the selection of confounders for OP among previous studies. Our findings, based on large-scale cohorts, suggested that greater exposure to night shift work is associated with an elevated risk of OP after adjusting for multiple variables. In addition, our findings, combined with previous studies, indicated that night shift work could potentially contribute to the risk of OP with fractures (31).

To our knowledge, this present study is the first one to investigate the link between lifetime night shift work, genetic susceptibility and osteoporosis with a large sample size. The study primarily focuses on three main aspects: the duration, frequency and length of night shifts. Our study reveals an increase in the likelihood of osteoporosis with a longer duration (>10 years) and frequency (3–8/month) after adjusting for multiple variables. These collective findings confirm that prolonged exposure to night shift work is indeed a risk factor for osteoporosis, which aligns with the findings of previous (32). Feskanich et al. (31) conducted a retrospective study on the prevalence of bone fractures in night shift nurses and found that working for more than 10 years significantly increases the risk of fractures. Another large-scale study reported a higher risk of atrial fibrillation and coronary heart disease in the night shift cohort with a duration of ≥10 years (11). Interestingly, no significant linear correlation was observed between the frequency of night shift work and the incidence rate of osteoporosis. In this cohort, with a night shift work frequency of 3–8 times per month, the risk of osteoporosis reached its highest level. Similar trends have also been reported in previous studies. Vetter et al. found that shorter lifetime exposure (<10 years) to night shifts correlated with higher diabetes risk (20). Wang et al. (11) revealed that individuals working night shifts 3–8 times per month had the highest risk of atrial fibrillation patients. These non-linear trends may be due to differences in study design or the healthy worker effect. In addition, night shift cohorts in UK Biobank might exhibit healthier lifestyles than day workers, like lower alcohol intake and higher physical activity, which might explain this results.

The underlying mechanism by which night shift work increases the risk of OP occurrence is still unclear, the disruption of circadian rhythms presents a conceivable factor contributing to the heightened risk of OP and OP-related pathological fracture. Irregular work schedules can disrupt the natural daily rhythm of hormone secretion, such as melatonin and cortisol, as well as impact the formation of bone turnover markers (BTMs) (33, 34). Melatonin, for instance, is recognized for its beneficial role in addressing menopause-related physical conditions and maintaining balanced bone remodeling to mitigate bone loss (35–37). The levels of bone turnover markers typically peak during the night and early morning, with disturbances to this pattern potentially inhibit proper bone remodeling processes (34). Additionally, night shift workers might experience reduced exposure to sunlight, resulting in lower levels of vitamin D, a crucial nutrient for optimal bone health (38, 39).

Stratified analyses were further taken to explore the potential interaction effects of confounding factors. Previous studies have indicated that night shifts can lead to circadian misalignment, with women possibly being more susceptible to changes in circadian rhythms (40, 41). Despite the stronger association between night shift work and OP risk in females, our study did not find a significant interaction effect between sex and the current or lifetime night shift work status, which need more evidence and rigorous exploration. Moreover, we also investigated the potential interaction between sleep chronotypes and night shift work, although without significant interactions, while comparing with “evening sleep chronotype,” the “morning or intermediate sleep chronotype” subgroups with an increasing night shift work length were positively associated with a higher risk of osteoporosis. This suggests that disruptions in the biological clock may play a significant role in the occurrence of osteoporosis, aligning with earlier research findings. As Phillips notes, the sleep duration of night shift workers without obviously decrease, the negative health effect might due to direct circadian misalignment and biological clock broken (42). Akimova et al. (43) also report the similar result in a 53,211 participants UK Biobank cohort, they found that the night owl was more suitable for night shift work, especially worked ≥45 h per week.

Additionally, we also investigate the relationship between work status and polygenetic risk score for OP risk. The findings highlight that regardless of an individual’s level of genetic predisposition (low, intermediate, and high), the association between night shift work and the risk of OP remains consistent, with no significant interaction observed. In essence, night shift rotation appears to be an independent risk factor for OP, distinct from genetic variations. However, it is important to acknowledge that this study predominantly focused on the white population (>90%) in the UK. For a more comprehensive understanding, further research is needed to explore the potential genetic susceptibility in diverse populations. Nevertheless, this finding preliminary recommended that reducing night shifts status might benefit for OP prevent regardless of the genetic susceptibility.

### Strengths and limitations

Our study conducted a large-scale prospective study on the effect of current and lifetime night shifts, as well as the genetic link, on the incidence of OP after adjusting for multiple variables. But several limitations also existed in this study. Firstly, the study design was observational in nature, precluding the establishment of causal relationships despite identifying a discernible correlation between night shifts and the incidence of OP and OP- related pathological fractures. Secondly, the utilization of a self-reported lifetime questionnaire, administered online by participants, introduces the possibility of retrospective bias and inherent classification errors. Thirdly, the observed incidence of OP in our study appeared comparatively lower when juxtaposed against previous research endeavors (e.g., NCHS). This discrepancy may stem from certain cases experiencing delayed diagnoses, thereby potentially underestimating the true incidence rate within our cohort. Fourthly, it is noteworthy that our OP cohort was predominantly composed of females. Given the well-documented increase in OP incidence among women above the age of 50, the sex distribution in our cohort might have influenced the observed outcomes. Fifthly, despite accounting for major osteoporosis risk factors, residual confounding cannot be fully excluded. Moreover, the details of the exposure or relevant variables provided in the database remains limited, for example, lack of occupational categories and work schedule transitions of night shift workers, or the quality of sleep and sleep associated disorder diagnosis exclude, which might influence the observed associations. Lastly, it is worth noting that the current and lifetime employment information was only assessed at the baseline, and it may have changed over the course of the study, potentially influencing the outcomes.

## Conclusion

In summary, our study illustrated the positive association between both current and lifetime night shift work status and the risk of OP and OP-related pathological fracture regardless of the genetic susceptibility. This study has public health implications regarding work involving night work and bone health.

## Data Availability

The original contributions presented in the study are included in the article/Supplementary material, further inquiries can be directed to the corresponding authors.

## Footnotes

1
https://www.ukbiobank.ac.uk/

2
https://biobank.ndph.ox.ac.uk/showcase/label.cgi?id=2413

3
https://github.com/Genomicsplc/ukb-pret
