# Supplementary material for: Association between night shift work and risk of osteoporosis and osteoporosis-related pathological fracture
Source: Front Public Health. 2026 Jan 13;13:1719807. doi: 10.3389/fpubh.2025.1719807 (PMC12835380; doi:10.3389/fpubh.2025.1719807)
Supplement: Supplementary file 1 [file Data_Sheet_1.docx]

**Appendix**

**Association between Night Shift Work and Risk of Osteoporosis and Osteoporosis-Related Pathological Fracture**

**Supplemental Figure 1** Flowchart of study process.

**Supplemental Table 1** UK Biobank participants' characteristics by the cumulative duration of lifetime exposure to all reported night shift work (n=75,120)

**Supplemental Table 2** UK Biobank participants' characteristics by the average frequency of lifetime night shift work exposure (n=75,120)

**Supplemental Table 3** Multivariable-adjusted HRs (95% CIs) for osteoporosis >2 years from the baseline (276,043)

**Supplemental Table 4** Current shift work and osteoporosis with fracture in the UK Biobank (n=276,774)

**Supplemental Table 5** Multivariable-adjusted HRs (95% CIs) for osteoporosis after multiple imputation

**Supplemental Table 6** Association between lifetime night shift across previous reported jobs and the risk of osteoporosis incident from the baseline after multiple imputation

**Supplemental Table 7** Current night shift work and osteoporosis odds after adjusted for other confounders

**Supplemental Table 8** Lifetime duration and frequency of work involving night shifts and osteoporosis odds after adjusted for other confounders

**Supplemental Table 9** Association between current night shift work and osteoporosis risk on the female-specific cohorts after additional adjusted for menopause and hormones use

**Supplemental Table 10** Lifetime night shift work and osteoporosis risk in female group after adjusted for menopause and hormone use

**Supplemental Table 11** Association of current shift work and osteoporosis HRs by sex, BMI, sleep duration, sleep chronotype and AAOS suggested index

**Supplemental Table 12** Association duration of lifetime work involving night shifts and osteoporosis HRs by sex, BMI, sleep duration, sleep chronotype and AAOS suggested index

**Supplemental Table 13** Association of average number of night shifts worked per month and osteoporosis HRs by sex, BMI, sleep duration, sleep chronotype and AAOS suggested index

**Supplemental Table 14** Multivariable-adjusted HRs (95% CI) for standard PRS tertliles and osteoporosis (n = 270,483)

**Supplemental Table 15** Multivariable-adjusted OR (95% CI) for standard PRS tertliles and osteoporosis incident at the baseline (n=73,561)

**Supplemental Table 16** Joint effect of lifetime cumulative duration of night shifts worked and PRS on the osteoporosis odds (n=73,561)

**Supplemental Table 17** Joint effect of average frequency of lifetime night shift worked and PRS on the risk of incident osteoporosis (n=73,561)

The primary outcome measure undertaken in this study was osteoporosis, further categorized into three distinct subtypes as per the 10th Revision of the International Classification of Diseases (ICD-10). These subtypes encompassed osteoporosis with pathological fracture (field 131963, ICD code M80), osteoporosis without pathological fracture (field 131965, ICD code M81), and osteoporosis in diseases classified elsewhere (field 131967, ICD code M82). Detailed information regarding the linkage procedure is available online (<https://biobank.ndph.ox.ac.uk/showcase/search.cgi?wot=0&srch=osteoporosis&yfirst=2000&ylast=2024>)

**UK Biobank cohort recruited individuals from 2006 ~ 2010 (n ~ 502,363)**

**(n ~ 502,363)**

**Participants with paid employment at baseline (n ~ 279,826)**

**(n ~ 502,363)**

**Exclusion: retired, unemployed or lacking important variables (n ~ 215,664)**

**(n ~ 502,363)**

**Prospective study(n ~ 276,774):**

**Current night shift work with OP**

**Cross-section study (n ~ 75,120):**

**Lifetime night shift work with OP**

**Exclusion: with prevalent OP at the baseline (n ~ 3,052)**

**Exclusion: without lifetime information (n ~ 204,706)**

**Night shift with**

**OP-related fracture**

**Night shift with**

**OP**

**Night shift duration**

**with OP**

**Night shift frequency**

**with OP**

**Genetic analysis**

**(n ~ 270,483)**

**Genetic analysis**

**(n ~ 73,561)**

**Supplemental Figure 1** Flowchart of study process. Abbreviations: OP, osteoporosis

**Supplemental Table 1** UK Biobank participants' characteristics by the cumulative duration of lifetime exposure to all reported night shift work (n=75,120)

|  | Current work schedule | | | |
| --- | --- | --- | --- | --- |
|  | None | < 5 years | 5 - 10 years | > 10 years |
| N (%) | 57,200 (76.1) | 6,914 (9.2) | 3,883 (5.2) | 7,123 (9.5) |
| Age (years) | 53.0 (6.8) | 52.5 (7.0) | 52.1 (6.9) | 52.7 (6.7) |
| Sex (male, %) | 41.7 | 54.3 | 48.8 | 59.2 |
| BMI (kg/m^2^) | 26.5 (4.5) | 27.2 (4.7) | 27.2 (4.7) | 27.9 (4.8) |
| Ethnicity (white, %) | 97.4 | 96.3 | 96.0 | 96.3 |
| Education (university or college, %) | 54.6 | 47.7 | 42.2 | 32.0 |
| Townsend deprivation index | -1.7 (2.8) | -1.5 (2.9) | -1.5 (2.9) | -1.6 (2.8) |
| Sleep duration (%) (h/day) | 7.1 (0.9) | 7.0 (1.0) | 7.0 (1.0) | 7.0 (1.1) |
| Chronotype (evening, %) | 9.6 | 10.3 | 10.4 | 11.7 |
| Smoke |  |  |  |  |
| Never | 62.2 | 54.7 | 54.8 | 53.8 |
| Previous | 31.1 | 36.6 | 35.3 | 36.6 |
| Current | 6.7 | 8.7 | 9.8 | 9.6 |
| Alcohol |  |  |  |  |
| Never | 2.4 | 1.8 | 2.6 | 2.6 |
| Previous | 2.2 | 2.5 | 2.7 | 2.9 |
| Current | 95.4 | 95.7 | 94.7 | 94.5 |
| Physical activity at goal |  |  |  |  |
| No | 19.1 | 17.5 | 16.1 | 15.0 |
| Yes | 68.3 | 71.0 | 71.1 | 69.6 |
| Missing | 12.7 | 11.6 | 12.7 | 15.4 |
| Healthy diet |  |  |  |  |
| Vegetable (tablespoons/ day) | 4.8 (3.1) | 4.8 (3.2) | 4.8 (2.8) | 4.8 (3.4) |
| Fruit (tablespoons/ day) | 3.1 (2.3) | 3.1 (2.4) | 3.1 (2.3) | 3.0 (2.5) |
| Fish (times/ week) | 2.1 (1.5) | 2.2 (1.5) | 2.1 (1.5) | 2.2 (1.5) |
| Processed meat (times/ week) | 1.4 (1.4) | 1.5 (1.4) | 1.5 (1.4) | 1.6 (1.4) |
| Unprocessed meat (times/ week) | 3.9 (2.0) | 4.0 (2.1) | 4.1 (2.2) | 4.2 (2.2) |
| Diabetes (%) | 2.2 | 3.2 | 3.6 | 3.4 |
| Cancer (%) | 5.8 | 5.1 | 5.2 | 5.3 |
| Summer outdoor time (hours/ per day) | 2.9 (2.0) | 3.2 (2.2) | 3.3 (2.3) | 3.7 (2.5) |
| Winter outdoor time (hours/ per day) | 1.4 (1.3) | 1.7 (1.6) | 1.7 (1.7) | 2.0 (2.0) |
| Mineral and other dietary supplements (%) | 37.5 | 35.4 | 35.7 | 36.6 |
| Vitamin D supplements (%) | 1.8 | 1.5 | 1.9 | 1.6 |

Continuous variables are displayed as means (SD), and categorical variables are presented as percentages.

Mineral and other Dietary supplements include fish oil, glucosamine, and calcium.

**Supplemental Table 2** UK Biobank participants' characteristics by the average frequency of lifetime night shift work exposure (n=75,120)

|  | Current work schedule | | | |
| --- | --- | --- | --- | --- |
|  | None | < 3/ months | 3 - 8/ months | > 8/ months |
| N (%) | 57,200 (76.1) | 2386 (3.2) | 8,946 (11.9) | 6,588 (8.8) |
| Age (years) | 53.0 (6.8) | 53.1 (6.9) | 51.9 (6.7) | 53.0 (6.9) |
| Sex (male, %) | 41.7 | 57.6 | 50.1 | 60.9 |
| BMI (kg/m^2^) | 26.5 (4.5) | 27.1 (4.6) | 27.2 (4.7) | 27.9 (4.8) |
| Ethnicity (white, %) | 97.4 | 95.1 | 96.4 | 96.4 |
| Education (university or college, %) | 54.6 | 45.6 | 42.3 | 35.7 |
| Townsend deprivation index | -1.7 (2.8) | -1.6 (2.8) | -1.6 (2.8) | -1.3 (3.0) |
| Sleep duration (%) (h/day) | 7.1 (0.9) | 7.0 (1.0) | 7.0 (1.0) | 7.0 (1.1) |
| Chronotype (evening, %) | 9.6 | 10.2 | 10.4 | 11.8 |
| Smoke |  |  |  |  |
| Never | 62.2 | 53.7 | 57.3 | 50.6 |
| Previous | 31.1 | 37.8 | 34.4 | 38.4 |
| Current | 6.7 | 8.5 | 8.2 | 11.0 |
| Alcohol |  |  |  |  |
| Never | 2.4 | 2.3 | 2.3 | 2.3 |
| Previous | 2.2 | 3.4 | 2.4 | 2.9 |
| Current | 95.4 | 94.3 | 95.3 | 94.9 |
| Physical activity at goal |  |  |  |  |
| No | 19.1 | 17.4 | 16.3 | 15.7 |
| Yes | 68.3 | 68.7 | 70.7 | 70.7 |
| Missing | 12.7 | 13.9 | 13.0 | 13.6 |
| Healthy diet |  |  |  |  |
| Vegetable (tablespoons/ day) | 4.8 (3.1) | 4.7 (3.0) | 4.8 (3.1) | 4.8 (3.4) |
| Fruit (tablespoons/ day) | 3.1 (2.3) | 3.1 (2.7) | 3.1 (2.3) | 3.0 (2.4) |
| Fish (times/ week) | 2.1 (1.5) | 2.1 (1.5) | 2.1 (1.5) | 2.2 (1.6) |
| Processed meat (times/ week) | 1.4 (1.4) | 1.5 (1.4) | 1.5 (1.4) | 1.6 (1.5) |
| Unprocessed meat (times/ week) | 3.9 (2.0) | 4.0 (2.1) | 4.1 (2.1) | 4.2 (2.2) |
| Diabetes (%) | 2.2 | 3.7 | 2.8 | 4.0 |
| Cancer (%) | 5.8 | 6.0 | 5.1 | 5.2 |
| Summer outdoor time (hours/ per day) | 2.9 (2.0) | 3.4 (2.3) | 3.3 (2.2) | 3.6 (2.5) |
| Winter outdoor time (hours/ per day) | 1.4 (1.3) | 1.8 (1.7) | 1.7 (1.6) | 2.0 (2.0) |
| Mineral and other dietary supplements (%) | 37.5 | 34.8 | 36.0 | 36.3 |
| Vitamin D supplements (%) | 1.8 | 1.7 | 1.7 | 1.5 |

Continuous variables are displayed as means (SD), and categorical variables are presented as percentages.

Mineral and other Dietary supplements include fish oil, glucosamine, and calcium.

**Supplemental Table 3** Multivariable-adjusted HRs (95% CIs) for osteoporosis >2 years from the baseline (276,391)

|  | Current work schedule | | | | *P* for trend |
| --- | --- | --- | --- | --- | --- |
|  | Day workers | Rarely night shifts | Some night shifts | Usual night shifts |  |
| Total cases | 4630 | 501 | 206 | 186 |  |
| Total sample size | 228,635 | 23,477 | 13,660 | 10,619 |  |
| Model 1 | 1.00 [reference] | 1.14 (1.04 - 1.25) | 1.17 (1.02 - 1.35) | 1.35 (1.16 - 1.56) | < 0.001 |
| Model 2 | 1.00 [reference] | 1.11 (1.01 - 1.22) | 1.15 (1.00 - 1.32) | 1.30 (1.12 - 1.51) | < 0.001 |
| Model 3 | 1.00 [reference] | 1.11 (1.00 - 1.22) | 1.10 (0.95 - 1.28) | 1.28 (1.10 - 1.50) | < 0.001 |

Model 1 adjusted for age, sex and BMI

Model 2 additionally adjusted for ethnicity (white, others), education (university or college, others) and Townsend deprivation index

Model 3 additionally adjusted for smoke (never, previous, current), alcohol (never, previous, current), physical activity at goal (no, yes, missing), healthy diet (vegetable, fruit, fish, unprocessed meat, processed meat)

**Supplemental Table 4** Current shift work and osteoporosis with fracture in the UK Biobank (n=276,774)

|  | Average length of night shift | | | *P* for trend |
| --- | --- | --- | --- | --- |
|  | None | Rarely/ some night shift | Usual night shift |  |
| Total cases | 336 | 52 | 22 |  |
| Total sample cases | 228,971 | 37,179 | 10,624 |  |
| Model 1 | 1.00 [reference] | 1.13 (0.84 - 1.51) | 2.03 (1.31 - 3.13) | 0.005 |
| Model 2 | 1.00 [reference] | 1.13 (0.84 - 1.52) | 2.06 (1.33 - 3.22) | 0.005 |
| Model 3 | 1.00 [reference] | 0.99 (0.72 - 1.35) | 1.87 (1.20 - 2.94) | 0.062 |

Model 1 adjusted for age, sex and BMI

Model 2 additionally adjusted for ethnicity (white, others), education (university or college, others) and Townsend deprivation index

Model 3 additionally adjusted for smoke (never, previous, current), alcohol (never, previous, current), physical activity at goal (no, yes, missing), healthy diet (vegetable, fruit, fish, unprocessed meat, processed meat)

**Supplemental Table 5** Multivariable-adjusted HRs (95% CIs) for osteoporosis after multiple imputation

|  | Current work schedule | | | | *P* for trend |
| --- | --- | --- | --- | --- | --- |
|  | Day workers | Rarely night shifts | Some night shifts | Usual night shifts |  |
| Total cases | 4,966 | 526 | 223 | 191 |  |
| Total sample size | 228,971 | 23,502 | 13,677 | 10,624 |  |
| Model 1 | 1.00 [reference] | 1.12 (1.02 - 1.23) | 1.19 (1.04 - 1.36) | 1.29 (1.12 - 1.50) | < 0.001 |
| Model 2 | 1.00 [reference] | 1.09 (1.00 - 1.20) | 1.17 (1.02 - 1.33) | 1.26 (1.09 - 1.46) | < 0.001 |
| Model 3 | 1.00 [reference] | 1.09 (0.99 - 1.19) | 1.16 (1.01 - 1.32) | 1.24 (1.07 - 1.44) | < 0.001 |

Model 1 adjusted for age, sex and BMI

Model 2 additionally adjusted for ethnicity (white, others), education (university or college, others) and Townsend deprivation index

Model 3 additionally adjusted for smoke (never, previous, current), alcohol (never, previous, current), physical activity at goal (no, yes, missing), healthy diet (vegetable, fruit, fish, unprocessed meat, processed meat)

**Supplemental Table 6** Association between lifetime night shift across previous reported jobs and the risk of osteoporosis incident from the baseline after multiple imputation

|  | Incident osteoporosis in night shifts OR(95% CI) | | | | *P* for trend |
| --- | --- | --- | --- | --- | --- |
| **Lifetime duration** | None | < 5 years | 5 - 10 years | > 10 years |  |
| Model 1 | 1.00 [reference] | 1.22 (0.95 - 1.57) | 1.18 (0.85 - 1.64) | 1.18 (0.91 - 1.55) | 0.082 |
| Model 2 | 1.00 [reference] | 1.20 (0.93 - 1.55) | 1.18 (0.85 - 1.63) | 1.18 (0.90 - 1.55) | 0.090 |
| Model 3 | 1.00 [reference] | 1.20 (0.93 - 1.55) | 1.18 (0.85 - 1.63) | 1.17 (0.90 - 1.54) | 0.100 |
| **Lifetime frequency** | None | < 3/ months | 3 - 8/ months | > 8/ months |  |
| Model 1 | 1.00 [reference] | 0.84 (0.52 - 1.37) | 1.37 (1.10 - 1.70) | 1.10 (0.83 - 1.45) | 0.044 |
| Model 2 | 1.00 [reference] | 0.83 (0.51 - 1.35) | 1.37 (1.10 - 1.70) | 1.08 (0.81 - 1.43) | 0.055 |
| Model 3 | 1.00 [reference] | 0.83 (0.51 - 1.35) | 1.37 (1.10 - 1.70) | 1.07 (0.81 - 1.42) | 0.061 |

Model 1 adjusted for age, sex and BMI

Model 2 additionally adjusted for ethnicity (white, others), education (university or college, others) and Townsend deprivation index

Model 3 additionally adjusted for smoke (never, previous, current), alcohol (never, previous, current), physical activity at goal (no, yes, missing), healthy diet (vegetable, fruit, fish, unprocessed meat, processed meat)

**Supplemental Table 7** Current night shift work and osteoporosis odds after adjusted for other confounders

|  | Current work schedule | | | | *P* for trend |
| --- | --- | --- | --- | --- | --- |
|  | Day workers | Rarely night shifts | Some night shifts | Usual night shifts |  |
| Model I | 1.00 [reference] | 1.09 (0.99 - 1.19) | 1.10 (0.95 - 1.26) | 1.23 (1.06 - 1.44) | 0.001 |
| Model II | 1.00 [reference] | 1.08 (0.98 - 1.19) | 1.12 (0.97 - 1.30) | 1.25 (1.06 - 1.46) | 0.001 |
| Model III | 1.00 [reference] | 1.06 (0.96 - 1.17) | 1.12 (0.97 - 1.30) | 1.20 (1.02 - 1.41) | 0.006 |
| Model IV | 1.00 [reference] | 1.09 (0.99 - 1.20) | 1.13 (0.98 - 1.30) | 1.24 (1.07 - 1.45) | < 0.001 |

Model I finial model additionally adjusted for chronic disease (diabetes) and cancer

Model II finial model additionally adjusted for sleep duration and sleep chronotype

Model III finial model additionally adjusted time spend outdoors in summer and winter

Model IV finial model additionally adjusted mineral and other dietary supplements (fish oil, glucosamine, calcium and others) and vitamin (vitamin D and others)

**Supplemental Table 8** Lifetime duration and frequency of work involving night shifts and osteoporosis odds after adjusted for other confounders

|  | Incident osteoporosis in night shifts ORs (95% CI) | | | | *P* for trend |
| --- | --- | --- | --- | --- | --- |
| **Lifetime duration** | None | < 5 years | 5 - 10 years | > 10 years |  |
| Model I | 1.00 [reference] | 1.17 (0.90 - 1.52) | 1.20 (0.86 - 1.66) | 1.19 (0.91 - 1.56) | 0.082 |
| Model II | 1.00 [reference] | 1.16 (0.89 - 1.52) | 1.21 (0.86 - 1.69) | 1.20 (0.91 - 1.58) | 0.086 |
| Model III | 1.00 [reference] | 1.25 (0.96 - 1.62) | 1.23 (0.88 - 1.71) | 1.13 (0.85 - 1.50) | 0.127 |
| Model IV | 1.00 [reference] | 1.19 (0.91 - 1.55) | 1.18 (0.85 - 1.64) | 1.19 (0.90 - 1.56) | 0.099 |
| **Lifetime frequency** | None | < 3/ months | 3 - 8/ months | > 8/ months |  |
| Model I | 1.00 [reference] | 0.78 (0.47 - 1.28) | 1.38 (1.11 - 1.72) | 1.07 (0.81 - 1.43) | 0.057 |
| Model II | 1.00 [reference] | 0.85 (0.51 - 1.41) | 1.32 (1.05 - 1.66) | 1.12 (0.84 - 1.50) | 0.062 |
| Model III | 1.00 [reference] | 0.90 (0.55 - 1.47) | 1.38 (1.10 - 1.72) | 1.07 (0.79 - 1.43) | 0.063 |
| Model IV | 1.00 [reference] | 0.86 (0.53 - 1.41) | 1.35 (1.08 - 1.68) | 1.07 (0.80 - 1.43) | 0.074 |

Model I finial model additionally adjusted for chronic disease (diabetes) and cancer

Model II finial model additionally adjusted for sleep duration and sleep chronotype

Model III finial model additionally adjusted time spend outdoors in summer and winter

Model IV finial model additionally adjusted mineral and other dietary supplements (fish oil, glucosamine, calcium and others) and vitamin (vitamin D and others)

**Supplemental Table 9** Association between current night shift work and osteoporosis risk on the female-specific cohorts after additional adjusted for menopause and hormones use

| Variable | HR (95%CI) |
| --- | --- |
| Current night shift work |  |
| Day workers | 1.00 [reference] |
| Rarely night shifts | 1.11 (1.00 - 1.22) |
| Some night shifts | 1.10 (0.93 - 1.29) |
| Usual night shifts | 1.26 (1.06 - 1.50) |
| *P* for trend | 0.002 |

**Supplemental Table 10** Lifetime night shift work and osteoporosis risk in female group after adjusted for menopause and hormone use

| Variable | OR (95%CI) | Variable | OR (95%CI) |
| --- | --- | --- | --- |
| Cumulative duration |  | Average frequency |  |
| None | 1.00 [reference] | None | 1.00 [reference] |
| < 5 years | 1.13 (0.83 - 1.52) | < 3/ months | 0.92 (0.53 - 1.57) |
| 5 - 10 years | 1.18 (0.82 - 1.70) | 3 - 8/ months | 1.40 (1.10 - 1.79) |
| > 10 years | 1.28 (0.95 - 1.73) | > 8/ months | 0.99 (0.70 - 1.39) |
| *P* for trend | 0.061 | *P* for trend | 0.128 |

**Supplemental Table 11** Association of current shift work and osteoporosis HRs by sex, BMI, sleep duration, sleep chronotype and AAOS suggested index

|  | Current work schedule | | | | *P* for interaction |
| --- | --- | --- | --- | --- | --- |
|  | Day workers | Rarely night shifts | Some night shifts | Usual night shifts |  |
| Sex^1^ |  |  |  |  |  |
| men | 1.00 [reference] | 0.97 (0.76 - 1.24) | 1.19 (0.91 - 1.57) | 1.09 (0.79 - 1.51) | 0.674 |
| women | 1.00 [reference] | 1.11 (1.01 - 1.23) | 1.10 (0.93 - 1.29) | 1.28 (1.08 - 1.52) |  |
| BMI^2^ |  |  |  |  |  |
| < 25.0 | 1.00 [reference] | 1.06 (0.92 - 1.21) | 1.15 (0.93 - 1.41) | 1.10 (0.86 - 1.41) | 0.614 |
| ≥ 25.0 | 1.00 [reference] | 1.10 (0.97 - 1.25) | 1.08 (0.89 - 1.31) | 1.28 (1.06 - 1.56) |  |
| Sleep chronotype^3^ |  |  |  |  |  |
| morning | 1.00 [reference] | 0.95 (0.78 - 1.14) | 1.39 (1.09 - 1.77) | 1.12 (0.81 - 1.55) | 0.156 |
| intermediate | 1.00 [reference] | 1.15 (1.02 - 1.30) | 1.02 (0.83 - 1.25) | 1.30 (1.05 - 1.60) |  |
| evening | 1.00 [reference] | 0.99 (0.70 - 1.39) | 0.92 (0.57 - 1.50) | 1.28 (0.88 - 1.86) |  |
| Sleep duration^3^ |  |  |  |  |  |
| < 8 h/ day | 1.00 [reference] | 1.14 (1.02 - 1.27) | 1.14 (0.96 - 1.34) | 1.32 (1.11 - 1.57) | 0.350 |
| ≥ 8 h/ day | 1.00 [reference] | 0.98 (0.82 - 1.17) | 1.11 (0.85 - 1.45) | 1.06 (0.79 - 1.44) |  |
| Smoke status^3^ |  |  |  |  |  |
| never | 1.00 [reference] | 1.07 (0.94 - 1.22) | 1.10 (0.90 - 1.35) | 1.20 (0.95 - 1.50) | 0.564 |
| current/ previous | 1.00 [reference] | 1.20 (0.98 - 1.28) | 1.17 (0.96 - 1.42) | 1.31 (1.07 - 1.61) |  |
| Alcohol status^3^ |  |  |  |  |  |
| never/ previous | 1.00 [reference] | 1.21 (0.90 - 1.62) | 1.22 (0.79 - 1.88) | 1.42 (0.93 - 2.16) | 0.876 |
| current | 1.00 [reference] | 1.08 (0.98 - 1.19) | 1.12 (0.97 - 1.30) | 1.22 (1.04 - 1.44) |  |
| Physical activity at goal^3^ |  |  |  |  |  |
| No | 1.00 [reference] | 1.03 (0.80 - 1.31) | 1.02 (0.67 - 1.54) | 1.27 (0.83 - 1.94) | 0.838 |
| Yes | 1.00 [reference] | 1.09 (0.97 - 1.23) | 1.21 (1.02 - 1.44) | 1.22 (1.00 - 1.49) |  |

Model 1 Adjusted for age, BMI, ethnicity (white/others), education (university or college / others), Townsend deprivation index and AAOS suggested index

Model 2 Adjusted for age, sex (men/ women), ethnicity (white/others), education (university or college / others), Townsend deprivation index and AAOS suggested index

Model 3 Adjusted for age, sex (men/ women), BMI, ethnicity (white/others), education (university or college / others), Townsend deprivation index and AAOS suggested index

**Supplemental Table 12** Association duration of lifetime work involving night shifts and osteoporosis HRs by sex, BMI, sleep duration, sleep chronotype and AAOS suggested index

|  | Current work schedule | | | | *P* for interaction |
| --- | --- | --- | --- | --- | --- |
|  | None | < 5 years | 5 - 10 years | > 10 years |  |
| Sex^1^ |  |  |  |  |  |
| men | 1.00 [reference] | 1.40 (0.82 - 2.37) | 1.18 (0.54 - 2.58) | 0.94 (0.50 - 1.76) | 0.673 |
| women | 1.00 [reference] | 1.19 (0.88 - 1.59) | 1.14 (0.79 - 1.65) | 1.31 (0.97 - 1.76) |  |
| BMI^2^ |  |  |  |  |  |
| < 25.0 | 1.00 [reference] | 1.12 (0.78 - 1.63) | 1.37 (0.90 - 2.10) | 1.49 (1.05 - 2.12) | 0.258 |
| ≥ 25.0 | 1.00 [reference] | 1.31 (0.92 - 1.88) | 0.90 (0.52 - 1.54) | 0.90 (0.59 - 1.36) |  |
| Sleep chronotype^3^ |  |  |  |  |  |
| morning | 1.00 [reference] | 0.82 (0.46 - 1.45) | 1.32 (0.73 - 2.41) | 1.30 (0.80 - 2.11) | 0.708 |
| intermediate | 1.00 [reference] | 1.34 (0.96 - 1.86) | 1.13 (0.72 - 1.76) | 1.16 (0.80 - 1.67) |  |
| evening | 1.00 [reference] | 1.61 (0.75 - 3.47) | 0.84 (0.25 - 2.82) | 1.20 (0.53 - 2.73) |  |
| Sleep duration^3^ |  |  |  |  |  |
| < 8 h/ day | 1.00 [reference] | 1.23 (0.90 - 1.66) | 1.10 (0.74 - 1.65) | 1.22 (0.89 - 1.67) | 0.971 |
| ≥ 8 h/ day | 1.00 [reference] | 1.23 (0.76 - 1.99) | 1.28 (0.71 - 2.32) | 1.20 (0.72 - 2.00) |  |
| Smoke status^3^ |  |  |  |  |  |
| never | 1.00 [reference] | 1.43 (1.03 - 1.99) | 1.02 (0.63 - 1.65) | 1.24 (0.86 - 1.79) | 0.454 |
| current/ previous | 1.00 [reference] | 1.00 (0.66 - 1.51) | 1.30 (0.82 - 2.08) | 1.18 (0.79 - 1.76) |  |
| Alcohol status^3^ |  |  |  |  |  |
| never/ previous | 1.00 [reference] | 1.03 (0.36 - 2.95) | 1.19 (0.36 - 3.98) | 1.63 (0.71 - 3.76) | 0.936 |
| current | 1.00 [reference] | 1.24 (0.95 - 1.62) | 1.15 (0.82 - 1.63) | 1.18 (0.89 - 1.57) |  |
| Physical activity at goal^3^ |  |  |  |  |  |
| No | 1.00 [reference] | 1.01 (0.51 - 2.02) | 0.77 (0.28 - 2.12) | 0.82 (0.36 - 1.90) | 0.752 |
| Yes | 1.00 [reference] | 1.26 (0.93 - 1.70) | 1.22 (0.83 - 1.81) | 1.20 (0.86 - 1.67) |  |

Model 1 Adjusted for age, BMI, ethnicity (white/others), education (university or college / others), Townsend deprivation index and AAOS suggested index

Model 2 Adjusted for age, sex (men/ women), ethnicity (white/others), education (university or college / others), Townsend deprivation index and AAOS suggested index

Model 3 Adjusted for age, sex (men/ women), BMI, ethnicity (white/others), education (university or college / others), Townsend deprivation index and AAOS suggested index

**Supplemental Table 13** Association of average number of night shifts worked per month and osteoporosis HRs by sex, BMI, sleep duration, sleep chronotype and AAOS suggested index

|  | Current work schedule | | | | *P* for interaction |
| --- | --- | --- | --- | --- | --- |
|  | None | < 3/ months | 3 - 8/ months | > 8/ months |  |
| Sex^1^ |  |  |  |  |  |
| men | 1.00 [reference] | 0.43 (0.10 - 1.74) | 1.28 (0.75 - 2.18) | 1.35 (0.79 - 2.33) | 0.462 |
| women | 1.00 [reference] | 0.92 (0.53 - 1.57) | 1.43 (1.12 - 1.82) | 1.02 (0.73 - 1.43) |  |
| BMI^2^ |  |  |  |  |  |
| < 25.0 | 1.00 [reference] | 0.92 (0.48 - 1.75) | 1.53 (1.14 - 2.05) | 1.15 (0.77 - 1.72) | 0.775 |
| ≥ 25.0 | 1.00 [reference] | 0.65 (0.29 - 1.46) | 1.21 (0.86 - 1.69) | 1.01 (0.67 - 1.51) |  |
| Sleep chronotype^3^ |  |  |  |  |  |
| morning | 1.00 [reference] | 0.89 (0.36 - 2.21) | 1.22 (0.79 - 1.89) | 1.04 (0.61 - 1.79) | 0.977 |
| intermediate | 1.00 [reference] | 0.70 (0.35 - 1.43) | 1.36 (1.01 - 1.82) | 1.25 (0.87 - 1.80) |  |
| evening | 1.00 [reference] | 1.00 (0.23 - 4.32) | 1.52 (0.75 - 3.09) | 1.05 (0.44 - 2.51) |  |
| Sleep duration^3^ |  |  |  |  |  |
| < 8 h/ day | 1.00 [reference] | 0.75 (0.41 - 1.37) | 1.25 (0.95 - 1.64) | 1.31 (0.95 - 1.79) | 0.085 |
| ≥ 8 h/ day | 1.00 [reference] | 0.94 (0.38 - 2.32) | 1.75 (1.20 - 2.55) | 0.63 (0.32 - 1.24) |  |
| Smoke^3^ |  |  |  |  |  |
| never | 1.00 [reference] | 0.65 (0.30 - 1.38) | 1.63 (1.24 - 2.16) | 0.97 (0.64 - 1.48) | 0.259 |
| current/ previous | 1.00 [reference] | 0.96 (0.49 - 1.89) | 1.11 (0.77 - 1.59) | 1.24 (0.84 - 1.83) |  |
| Alcohol^3^ |  |  |  |  |  |
| never/ previous | 1.00 [reference] | 2.07 (0.70 - 6.09) | 1.32 (0.55 - 3.17) | 0.94 (0.33 - 2.72) | 0.199 |
| current | 1.00 [reference] | 0.66 (0.37 - 1.17) | 1.40 (1.11 - 1.75) | 1.13 (0.84 - 1.52) |  |
| Physical activity at goal^3^ |  |  |  |  |  |
| No | 1.00 [reference] | 0.89 (0.28 - 2.88) | 0.58 (0.25 - 1.33) | 1.32 (0.68 - 2.58) | 0.181 |
| Yes | 1.00 [reference] | 0.79 (0.43 - 1.46) | 1.44 (1.11 - 1.87) | 1.10 (0.78 - 1.56) |  |

Model 1 Adjusted for age, BMI, ethnicity (white/others), education (university or college / others), Townsend deprivation index and AAOS suggested index

Model 2 Adjusted for age, sex (men/ women), ethnicity (white/others), education (university or college / others), Townsend deprivation index and AAOS suggested index

Model 3 Adjusted for age, sex (men/ women), BMI, ethnicity (white/others), education (university or college / others), Townsend deprivation index and AAOS suggested index

**Supplemental Table 14** Multivariable-adjusted HRs (95% CI) for standard PRS tertliles and osteoporosis (n = 270,483)

| PRS tertiles | Total cases | Total sample size | HRs (95% CI) |
| --- | --- | --- | --- |
| Low (~ 33.3%) | 1,193 | 90,161 | 1.00 [reference] |
| Intermediate (33.3%~66.7%) | 1,882 | 90,161 | 1.58 (1.47 - 1.70) |
| High (66.7% ~) | 2,663 | 90,161 | 2.31 (2.15 - 2.48) |

Adjusted for age, sex, BMI, ethnicity (white, others), education (university or college, others), Townsend deprivation index, smoke (never, previous, current), alcohol (never, previous, current), physical activity at goal (no, yes, missing), healthy diet (vegetable, fruit, fish, unprocessed meat, processed meat)

**Supplemental Table 15** Multivariable-adjusted OR (95% CI) for standard PRS tertliles and osteoporosis incident at the baseline (n=73,561)

| PRS tertiles | Total cases | Total sample size | OR (95% CI) |
| --- | --- | --- | --- |
| Low (~ 33.3%) | 161 | 24,520 | 1.00 [reference] |
| Intermediate (33.3%~66.7%) | 246 | 24,520 | 1.51 (1.23 - 1.85) |
| High (66.7% ~) | 377 | 24,521 | 2.42 (2.00 - 2.92) |

Adjusted for age, sex, BMI, ethnicity (white, others), education (university or college, others), Townsend deprivation index, smoke (never, previous, current), alcohol (never, previous, current), physical activity at goal (no, yes, missing), healthy diet (vegetable, fruit, fish, unprocessed meat, processed meat)

**Supplemental Table 16** Joint effect of lifetime cumulative duration of night shifts worked and PRS on the osteoporosis odds (n=73,561)

| Subgroup | OR (95% CI) | P for interaction |
| --- | --- | --- |
| Low genetic risk (~ 33.3%) |  | 0.601 |
| Day workers | 1.00 [reference] |  |
| < 5 years | 1.46 (0.87 - 2.48) |  |
| 5 - 10 years  > 10 years | 1.05 (0.49 - 2.26) |  |
|  | 0.83 (0.40 - 1.71) |  |
| Intermediate genetic risk (33.3%~66.7%) |  |  |
| Day workers | 1.50 (1.19 - 1.88) |  |
| < 5 years | 1.87 (1.17 - 2.99) |  |
| 5 - 10 years  > 10 years | 1.31 (0.66 - 2.60) |  |
|  | 1.90 (1.19 - 3.03) |  |
| High genetic risk (66.7% ~) |  |  |
| Day workers | 2.36 (1.91 - 2.92) |  |
| < 5 years | 2.50 (1.65 - 3.80) |  |
| 5 - 10 years  > 10 years | 3.43 (2.16 - 5.46) |  |
|  | 3.35 (2.25 - 4.98) |  |

Adjusted for age, sex, BMI, ethnicity (white, others), education (university or college, others), Townsend deprivation index, smoke (never, previous, current), alcohol (never, previous, current), physical activity at goal (no, yes, missing), healthy diet (vegetable, fruit, fish, unprocessed meat, processed meat)

**Supplemental Table 17** Joint effect of average frequency of lifetime night shift worked and PRS on the risk of incident osteoporosis (n=73,561)

| Subgroup | OR (95% CI) | P for interaction |
| --- | --- | --- |
| Low genetic risk (~ 33.3%) |  | 0.424 |
| Day workers | 1.00 [reference] |  |
| < 3/ months | 1.29 (0.52 - 3.19) |  |
| 3 - 8/ months  > 8/ months | 1.22 (0.73 - 2.03) |  |
|  | 0.96 (0.48 - 1.89) |  |
| Intermediate genetic risk (33.3%~66.7%) |  |  |
| Day workers | 1.50 (1.19 - 1.88) |  |
| < 3/ months | 1.82 (0.88 - 3.77) |  |
| 3 - 8/ months  > 8/ months | 1.91 (1.26 - 2.92) |  |
|  | 1.50 (0.88 - 2.54) |  |
| High genetic risk (66.7% ~) |  |  |
| Day workers | 2.36 (1.91 - 2.92) |  |
| < 3/ months | 0.78 (0.25 - 2.46) |  |
| 3 - 8/ months  > 8/ months | 3.67 (2.63 - 5.12) |  |
|  | 2.96 (1.95 - 4.51) |  |

Adjusted for age, sex, BMI, ethnicity (white, others), education (university or college, others), Townsend deprivation index, smoke (never, previous, current), alcohol (never, previous, current), physical activity at goal (no, yes, missing), healthy diet (vegetable, fruit, fish, unprocessed meat, processed meat)
