# Supplementary material for: Association between night shift work and risk of osteoporosis and osteoporosis-related pathological fracture
Source: Front Public Health. 2026 Jan 13;13:1719807. doi: 10.3389/fpubh.2025.1719807 (PMC12835380; doi:10.3389/fpubh.2025.1719807)
Supplement: Supplementary file 2 [file Data_Sheet_2.PDF]

STROBE Statement—checklist of items that should be included in reports of observational studies

	Item No.	Recommendation	Page No.	Relevant text from manuscript
**Title and abstract**	1	(*a*) Indicate the study's design with a commonly used term in the title or the abstract	2	The multivariable-adjusted logistic regression and Cox proportional hazard models were utilized to analyze the potential links between night shift work and the incidence of OP.
		(*b*) Provide in the abstract an informative and balanced summary of what was done and what was found	2	This study demonstrated that both current and lifetime night shifts was significant associated with the greater risk of OP and its related pathological fracture. Additionally, these associations were not modified by genetic susceptibility.
**Introduction**				
Background/rationale	2	Explain the scientific background and rationale for the investigation being reported	3	In recent years, numerous researches indicated that night shifts could increase the risk of disease incidence and health damage, such as obesity, healthy aging and coronary heart disease, only several small-scale investigations evidence involved OP, and the correlation between night shift work and OP has remained inconclusive.
Objectives	3	State specific objectives, including any prespecified hypotheses	3	Our primary research objective is to analyze the potential linkage between current night shift work and an elevated risk of developing OP. Furthermore, an exploration into the interplay between night shift work and genetic predisposition to OP was undertaken to ascertain its impact on disease susceptibility. we also evaluated the association between night shift work and the risk of OP-related pathological fractures.
**Methods**				
Study design	4	Present key elements of study design early in the paper	6-8	A Cox proportional hazards model
Setting	5	Describe the setting, locations, and relevant dates, including periods of recruitment, exposure, follow-up, and data collection	4	During the period of 2006 to 2010, a prospective cohort study in the United Kingdom (UK), UK biobank, enlisted over half a million individuals. The participants, spanning ages 37 to 73, data compilation encompassing a spectrum of facets, spanning lifestyle, mental well-being, physical metrics, biological evaluations, and genomic information
Participants	6	(*a*) *Cohort study*—Give the eligibility criteria, and the sources and methods of selection of	4	Accordingly, a total of 276,774 participants were included in the main analysis. In the prospective study within the current night shift cohorts, individuals with a pre-existing diagnosis of osteoporosis were excluded from consideration (N=3,052).

		participants. Describe methods of follow-up<br>*Case-control study*—Give the eligibility criteria, and the sources and methods of case ascertainment and control selection. Give the rationale for the choice of cases and controls		In addition, the UK Biobank program conducted an online lifetime employment questionnaire follow-up with approximately 330,000 participants who had provided contactable email addresses, regardless of their work status at the baseline assessment. Among them, a subset was conformed to our inclusion criteria (N=75,120)
		(*b*) *Cohort study*—For matched studies, give matching criteria and number of exposed and unexposed		NA
Variables	7	Clearly define all outcomes, exposures, predictors, potential confounders, and effect modifiers. Give diagnostic criteria, if applicable	5	Outcome: osteoporosis<br>Exposure: night shift work<br>Confounders: age, sex, BMI, education, ethnicity, Townsend index, smoke status, alcohol status, physical activity, health diet, sleep chronotype and duration and additional diabetes, calcium and vitamin D supplements, menopause and hormone use (female-specific group). chronic diseases (diabetes) and cancer<br>Effect modifiers: PRS
Data sources/ measurement	8*	For each variable of interest, give sources of data and details of methods of assessment (measurement). Describe comparability of assessment methods if there is more than one group	6	The measurement of current and lifetime night shift work<br>Assessment of outcomes<br>PRS<br>Covariates
Bias	9	Describe any efforts to address potential sources of bias	7	Several sensitive analyses were conducted in our study: 1) excluded participants who occurred osteoporosis within the first 2 years after the recruitment; ...... 7) In female-specific group, additional adjust for menopause and hormones use. .
Study size	10	Explain how the study size was arrived at	4	In the prospective study within the current night shift cohorts, individuals with a pre-existing diagnosis of osteoporosis were excluded from consideration
Quantitative variables	11	Explain how quantitativeariables were handled in the analyses. If applicable, describe which groupings were chosen and why	6	moderate activities ≥150 min/week and/or vigorous activity ≥75 min/week, or equivalent combination aerobic according to the activity guideline of the UK, quantified in metabolic equivalent task (MET-hours/week).

Statistical methods	12	(*a*) Describe all statistical methods, including those used to control for confounding	6-8	The Cox proportional hazard models were used to calculate the hazard ratios (HR) and 95% confidence intervals (CI) in the association between current night shift and incident osteoporosis. Adjustment for age, sex, ethnicity, education, and the Townsend index, and AAOS OP-relevant variables.
		(*b*) Describe any methods used to examine subgroups and interactions	7	We use the interaction analysis based on a log likelihood ratio test.
		(*c*) Explain how missing data were addressed	6-8	Subjects without complete data on night shift work and important covariates were excluded. Categorical variables, such as physical activity, were treated as missing indicator categories.
		(*d*) *Cohort study*—If applicable, explain how loss to follow-up was addressed<br>*Case-control study*—If applicable, explain how matching of cases and controls was addressed<br>*Cross-sectional study*—If applicable, describe analytical methods taking account of sampling strategy		N/A
		(*e*) Describe any sensitivity analyses	7	Several sensitive analyses were conducted in our study: 1) excluded participants who occurred osteoporosis within the first 2 years after the recruitment;
Results				
Participants	13*	(a) Report numbers of individuals at each stage of study—eg numbers potentially eligible, examined for eligibility, confirmed eligible, included in the study, completing follow-up, and analysed	9	Recruited into UK Biobank cohorts: ~500,000 subjects<br>Complete the occupational information: ~276,774 subjects; 5,906 occurrences of OP events<br>With lifetime work history complete data: 75,120 participates with 806 OP cases.
		(b) Give reasons for non-participation at each stage	9	Subjects with osteoporosis at baseline or miss data in important covariates.
		(c) Consider use of a flow diagram	4	Supplementary figure 1
Descriptive data	14*	(a) Give characteristics of study participants (eg demographic, clinical, social) and information on exposures and potential confounders	9	The demographic characteristics and potential confounders are shown in Table 1 and Supplementary Table 1-2.
		(b) Indicate number of participants with missing data for each variable of interest	9	The demographic characteristics and potential confounders are shown in Table 1.
		(c) *Cohort study*—Summarise follow-up time (eg,	9	The median follow-up was 13.7 years.

		average and total amount)		
Outcome data	15*	*Cohort study*—Report numbers of outcome events or summary measures over time	9	The demographic characteristics and potential confounders are shown in Table 1.
		Case-control study—Report numbers in each exposure category, or summary measures of exposure		
		Cross-sectional study—Report numbers of outcome events or summary measures		
Main results	16	(*a*) Give unadjusted estimates and, if applicable, confounder-adjusted estimates and their precision (eg, 95% confidence interval). Make clear which confounders were adjusted for and why they were included	9-10	participants who worked schedules involving night shifts for < 5 years had a higher likelihood of osteoporosis (OP) than those who never worked night shifts [OR 1.22, 95% CI (0.95 - 1.57)]. In the '>10 years' night shift exposure group have the highest OP risk [OR 1.21, 95% CI (0.92 - 1.58)]. In addition, participants who worked an average of 3–8 nights per month, rather than >8 nights per month, had a notable and higher risk of OP than those who never worked night shifts [OR 1.38, 95% CI (1.11–1.72)].
Other analyses	17	Report other analyses done—eg analyses of subgroups and interactions, and sensitivity analyses	10-11	Multivariable-adjusted HRs (95% CIs) for osteoporosis >2 years from the baseline; Current shift work and osteoporosis with fracture; Association duration of lifetime work involving night shifts and osteoporosis HRs by sex, BMI, sleep duration, sleep chronotype and AAOS suggested index
Discussion				
Key results	18	Summarise key results with reference to study objectives	12-13	In this large prospective study of over 280,000 individuals with about 6,000 OP cases in the UK Biobank, we observed that 1) current night shift workers showed an increased susceptibility to OP compared to those "day worker" after adjusting for other established risk factors. As the categories of night shift work increased (from "rare night shift" to "permanent night shift"), the trend in the incidence of OP became more pronounced; 2) Individuals exposed to night shift work for a lifetime duration exceeding 10 years or 3-8 per month shifts faced higher risk of developing OP; 3) no significant interaction was observed between night shift work in current and lifetime and genetic susceptibility to OP; 4) Besides, within current night shift cohort, the risk of suffering from OP with fractures appeared to be more prevalent in usually night shift worker..

Limitations	19	Discuss limitations of the study, taking into account sources of potential bias or imprecision. Discuss both direction and magnitude of any potential bias	14-15	Firstly, the study design was observational in nature, precluding the establishment of causal relationships despite identifying a discernible correlation between night shifts and the incidence of OP and OP- related pathological fractures ..... Fifthly, despite accounting for major osteoporosis risk factors, residual confounding cannot be fully excluded. Moreover, the details of the exposure or relevant variables provided in the database remains limited, for example, lack of occupational categories and work schedule transitions of night shift workers, or the quality of sleep and sleep associated disorder diagnosis exclude, which might influence the observed associations. Lastly, it is worth noting that the current and lifetime employment information was only assessed at the baseline, and it may have changed over the course of the study, potentially influencing the outcomes..
Interpretation	20	Give a cautious overall interpretation of results considering objectives, limitations, multiplicity of analyses, results from similar studies, and other relevant evidence	15	our study illustrated the positive association between both current and lifetime night shift work status and the risk of OP and OP-related pathological fracture regardless of the genetic susceptibility. This study has public health implications regarding work involving night work and bone health
Generalisability	21	Discuss the generalisability (external validity) of the study results	5	The potential of reducing night shift work as a strategy for preventing osteoporosis requires further investigation.
Other information				
Funding	22	Give the source of funding and the role of the funders for the present study and, if applicable, for the original study on which the present article is based	15	This research was funded by the National Natural Science Foundation of China, grant number No.82371600 the Natural Science Foundation of Hunan Province of Outstanding Young Scholars, grant number No.2021JJ20086, the Scientific Research Projects of the Health Commission of Hunan Province, project number No. B202304077119, and the Fundamental Research Funds for the Central Universities of Central South University, grant number No.2023ZZTS0558.

*Give information separately for cases and controls in case-control studies and, if applicable, for exposed and unexposed groups in cohort and cross-sectional studies.

Note: An Explanation and Elaboration article discusses each checklist item and gives methodological background and published examples of transparent reporting. The STROBE checklist is best used in conjunction with this article (freely available on the Web sites of PLoS Medicine at http://www.plosmedicine.org/, Annals of Internal Medicine at http://www.annals.org/, and Epidemiology at http://www.epidem.com/). Information on the STROBE Initiative is available at www.strobe-statement.org.
